# The Sound Sensation of Apical Electric Stimulation in Cochlear Implant Recipients with Contralateral Residual Hearing

**DOI:** 10.1371/journal.pone.0038687

**Published:** 2012-06-19

**Authors:** Diane S. Lazard, Jeremy Marozeau, Hugh J. McDermott

**Affiliations:** 1 Bionics Institute, East Melbourne, Victoria, Australia; 2 Department of Otolaryngology, The University of Melbourne, Melbourne, Victoria, Australia; University of Salamanca- Institute for Neuroscience of Castille and Leon and Medical School, Spain

## Abstract

**Background:**

Studies using vocoders as acoustic simulators of cochlear implants have generally focused on simulation of speech understanding, gender recognition, or music appreciation. The aim of the present experiment was to study the auditory sensation perceived by cochlear implant (CI) recipients with steady electrical stimulation on the most-apical electrode.

**Methodology/Principal Findings:**

Five unilateral CI users with contralateral residual hearing were asked to vary the parameters of an acoustic signal played to the non-implanted ear, in order to match its sensation to that of the electric stimulus. They also provided a rating of similarity between each acoustic sound they selected and the electric stimulus. On average across subjects, the sound rated as most similar was a complex signal with a concentration of energy around 523 Hz. This sound was inharmonic in 3 out of 5 subjects with a moderate, progressive increase in the spacing between the frequency components.

**Conclusions/Significance:**

For these subjects, the sound sensation created by steady electric stimulation on the most-apical electrode was neither a white noise nor a pure tone, but a complex signal with a progressive increase in the spacing between the frequency components in 3 out of 5 subjects. Knowing whether the inharmonic nature of the sound was related to the fact that the non-implanted ear was impaired has to be explored in single-sided deafened patients with a contralateral CI. These results may be used in the future to better understand peripheral and central auditory processing in relation to cochlear implants.

## Introduction

Cochlear implants (CIs) restore speech understanding by directly stimulating the spiral ganglion cells within the cochlea using electric pulse trains. In order to gain insight into the relationship between electric stimulation patterns and speech understanding of CI users, acoustic simulators of CIs have been developed [1], [2]. Typical simulators function similarly to CI sound processors, which filter acoustic signals into a number of frequency bands, and then extract the envelopes of the band-passed waveforms [3]. For each frequency band, the short-term envelope level is converted into the amplitude of electric pulses applied to the electrode corresponding to that band. Similarly, CI acoustic simulators divide the input signal into a number of frequency channels by means of band-pass filters, and extract the temporal envelopes. These envelopes are used to modulate a set of carrier signals which are finally summed to produce a composite acoustic waveform. Different types of carrier signals have been used in previously reported acoustic simulators, including pulse trains [4], harmonic complexes [5], pure tones [6], and noise bands [2]. Noise-band carriers are the most commonly used because they seem to provide the most accurate simulation for speech intelligibility modeling [7]. However, some researchers have found that existing simulators are not always accurate models of phoneme perception [8], and they may not reproduce exactly the sound perceived by CI users [7].

Several authors have investigated pitch matching between electric stimulation and acoustic sounds played to the non-implanted ear where residual hearing was present (e.g., [9]–[15]). However, Eddington et al [15], when testing a subject with normal contralateral hearing thresholds in the low frequencies up to 1000 Hz, found that pitch matching with a pure tone was difficult. Those authors hypothesized that the sounds heard by the implanted subjects were not pure tones, and that “to determine what subjects hear, it will be necessary to compare complex acoustic stimuli having a variety of spectral characteristics” [15]. Since then, no reports appear to have been published specifically about subjective quality or timbre comparisons between electric sensations and a range of complex sounds. Most studies about sound quality with a CI have focused on the evaluation of the perceived features of certain complex sounds such as speaker gender recognition [16], [17] or music appreciation [18]–[21]. These two listening experiences require different levels of auditory processing, involving high cognitive processes [22]. Therefore, the aim of the present study was to investigate more thoroughly the sound sensation evoked by the simplest pattern of electric stimulation, before considering more complex auditory stimuli.

Sets of acoustic stimuli covering a possible range of properties that might match the quality of electric stimulation were tested. Electric stimulation was generated on the most-apical electrode of a CI in implant users with residual hearing in the ear contralateral to the CI. The apical position of the electrode was expected to correspond well to the cochlear region where these subjects had most residual hearing. The subjects were asked to adjust complex acoustic sounds, played to the ear with residual hearing, to match as closely as possible the auditory sensation they perceived from the electric stimulus.

## Methods

### 1. Subjects

Five post-linguistically deaf adults with residual hearing in the non-implanted ear participated. They were recruited from the Cochlear Implant Clinic of the Royal Victorian Eye and Ear Hospital (East Melbourne, Australia). This project conformed to The Code of Ethics of the World Medical Association (Declaration of Helsinki), and was approved by the Royal Victorian Eye and Ear Hospital Human Research Ethics Committee (Project 10-995H). Each subject signed a written informed consent form. Subjects’ demographic and audiometric details for the non-implanted ear are given in Table S1 and Figure S1. Residual hearing thresholds were better than or equal to 75 dB HL at 500 Hz (average 65 dB HL), and 95 dB HL at 1000 Hz (average 80 dB HL). All subjects were experienced users (at least 1 year) of Cochlear® devices with the ACE sound-processing strategy. Representative speech-recognition scores in quiet using the CI alone (monaural condition), tested with consonant-nucleus-consonant (CNC) monosyllabic words, are provided in Table S1.

### 2. Stimuli

All auditory stimuli were created using the software MAX/MSP 5 (Cycling ’74 ®), which also provided the experimental interface and enabled data collection. The electric stimulus, which was delivered by electrode 22, was a pulse train with an overall duration of 710 ms, including a 10-ms ramp up and a 200-ms ramp down in level. It was similar to stimuli produced by the ACE strategy, with biphasic pulses having 25 µs per phase and an interphase gap of 8 µs. For each subject, the C- and T-levels and pulse rates used were those programmed for everyday use in their own sound processors. For all subjects except S3 (who had an older CI processor), the frequency band assigned to electrode 22 encompassed 188–313 Hz. For S3, this frequency band encompassed 120–280 Hz.

Acoustic stimuli were presented via insert earphones (Etymotic®, ER-4P). The temporal envelope was similar to that of the electric stimulus. Frequency-shaped amplification, with gains derived from the National Acoustic Laboratories’ NAL-RP formula [23], was applied according to each subject’s audiogram. A graphical interface (Bamboo Fun pen, Wacom®) was used by each subject to adjust acoustic signal parameters within a multi-dimensional space (see Figure S2A). The position of the pen (on virtual x and y axes) varied two selected parameters simultaneously as described below, while a slider on the side controlled the loudness of the acoustic signal. Three different types of sounds were presented in the experiments as described next and illustrated in Figure S2B. These signals were chosen because the findings of previous studies suggested they were likely to be perceived as similar to constant-rate stimulation on one electrode [8].

#### 2.1 White noise filtered through a band-pass filter (Condition 1)

A white noise was filtered through a fourth-order Butterworth filter. Each listener could vary the following parameters: one axis controlled the center frequency of the filter (ranging from 89 to 1264 Hz on a logarithmic scale), while the other axis controlled the Q factor of the filter (ranging from 300 to 0.15 on a logarithmic scale). The Q factor characterizes the bandwidth (Δf) of the filter relative to its center frequency (F0):
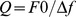
Therefore, a high Q value results in a relatively tonal sound, whereas a low Q results in a sound more similar to the original white noise.

#### 2.2 Harmonic complex sound with band-pass filtering (Condition 2)

An 11-harmonic complex sound was generated. Its fundamental frequency (F0) was equal to the center frequency selected by each subject at the end of testing Condition 1 (C1). This sound was filtered through an output filter, with parameters that could be modified by each subject. One axis controlled the center frequency of the filter (ranging from 40 to 22050 Hz on a logarithmic scale, 22050 Hz being half the sampling rate), while the other axis controlled the Q factor of this filter (ranging from 0.15 to 300 on a logarithmic scale). If the center frequency of the filter was set below the F0, the filter acted as a low-pass filter. Conversely, if the center frequency was set above the highest harmonic (11 × F0), the filter acted as a high-pass filter. Therefore, the effective width of the filter could affect the number of audible harmonics.

#### 2.3 Inharmonic complex sound with band-pass filtering (Condition 3)

An 11-component complex sound was generated and filtered through the output filter selected by each subject at the end of testing C2. Using the graphical interface, each listener could vary the following parameters: one axis controlled the F0 of the sound (ranging from 89 to 1264 Hz on a logarithmic scale), while the other axis controlled a parameter referred to as inharmonicity. The composite acoustic signal comprised components with frequencies defined by:
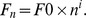
where F*_n_* was the frequency of each component (i.e., n was numbered 1–11), and i was the inharmonicity exponent, ranging from 0 to 2.8 on a linear scale. When i = 1 or 2, the sound was harmonic. Values of i lower than 1 resulted in a compression of the inter-component frequency spacing whereas values higher than 1 resulted in an expansion of the inter-component spacing. An example spectrum corresponding to the latter condition is illustrated in Figure S2B (lower right panel).

### 3. Procedure

First, the presentation level of the electric stimulus was set to be comfortable for each listener. The acoustic signal was then adjusted in level to match approximately the loudness of the electric stimulus; the resulting overall level at the eardrum was estimated to be around 90 dB SPL. The level of the acoustic signal could be modified by subjects during the experiment if variations to other parameters induced changes in loudness. The electric and acoustic signals were presented alternately to each ear.

Subjects were first familiarized with the interface. They were trained by a simple pitch-matching task with a pure tone played to the non-implanted ear (data not shown). Subsequently, conditions C1, C2, and C3 were presented in that order, and repeated 4 times in total. In order to reduce any tendency of subjects to return to the same spatial position on the interface and thereby bias the results, the settings of the interface were modified before each trial of each condition by interchanging the axes (x becoming y and *vice versa*), and by adding offsets to the origin of the axes (20% shift on each axis). At the beginning of each trial, the subjects could select any place on the tablet. The subjects were instructed to adjust the acoustic sound to make it as similar as possible to the perceived electric sensation. There was no time limitation, although the duration of each trial was recorded. Subjects were encouraged to explore the whole graphical interface to evaluate the range of acoustic possibilities. When the subjects reached the optimal match for one trial, the acoustic properties of the sound thus created were recorded by the software. One such sound was recorded per trial.

After each trial of each condition, subjects were asked to rate the similarity between the acoustic sound they had selected and the electric stimulus. Their responses were recorded on a line scale of 20 cm marked with “completely different” at one end and “exactly the same” at the other end. A number between 0 and 10 was assigned to the response, with 10 corresponding to “exactly the same”.

## Results

For each subject, the experimental data were calculated as the means of the responses from the 4 trials in each condition. Geometric means were used for frequencies and Q factors, and arithmetic averages were used for the inharmonicity exponents and the similarity ratings. The results are shown in Figure S3.

The average time for patients to perform one trial ranged from 1.5 to 3.25 minutes for condition C1, from 1.25 to 2.75 minutes for condition C2, and from 1.25 to 2.5 minutes for condition C3.

In condition C1 (Figure S3A), where the center frequency and bandwidth of a filtered noise were adjusted, the mean center frequency was 365 Hz (range of the individual responses was 192–710 Hz, range of the means across subjects was 266–482). The Q factor varied widely across subjects, from 1.0 to 348.3 for the individual responses (average 20.2, range of the means across subjects 4.6–106.6).

In condition C2 (Figure S3B), where the center frequency and bandwidth of a filtered 11-harmonic complex sound were adjusted, the parameters of the sounds selected were more similar than in condition C1. The average Q factor selected by the 5 subjects was 13.4 (range of the individual responses 1.27–146.1, range of the means across subjects 7.4–20.5). The center frequency of the filter (average 523 Hz, range of the individual responses 143–1970, range of the means across subjects 213–1462) was more variable across subjects than the Q factor, but the ratios between the mean center frequency selected in C1 and the mean center frequency selected in C2 were generally close to 1 (for subjects S1 to S5, respectively: 1.3, 1.1, 1.5, 0.8, 3.5).

Results from condition C3, in which the fundamental frequency of an 11-component complex sound and the inharmonicity exponent were varied, are shown in Figure S3C. If a component frequency was set higher than half the sampling rate, it was automatically “fold down” to a lower value. This aliasing effect appeared in two trials (trials 2 and 4 of S5) out of the twenty trials of C3. These two trials were consequently discarded from the analysis. Across all 5 subjects, the mean fundamental frequency was 285 Hz (range of the individual responses 115–659, range of the means across subjects 212–508), and the mean value for the inharmonicity exponent was 1.38 (range of the individual responses 0.34–2.15, range of the means across subjects 1.12–1.68).

The mean rating of the similarity between the acoustic sound subjects had selected and the electric stimulus was 4.6 for condition C1 (range of the individual responses 1–8, range of the means across subjects 2.75–6.5). Subjects S2 and S4 gave the lowest mean similarity ratings for this condition (4.0 and 2.75 respectively); they also selected the two extreme values (average and absolute values) of the Q factor among the 5 subjects in this condition. For condition C2, the mean rating was 7.3 (range of the individual responses 4–9.7, range of the means across subjects 5.4–8.4). Two subjects, S2 and S4, rated higher this condition than conditions C1 and C3. For condition C3, the mean rating was 7.5 (range of the individual responses 5–9.5, range of the means across subjects 6.50–9.1). Two subjects, S3 and S5, rated higher this last condition. On average, subject S1 rated similarly condition C2 and C3 (7.0 versus 7.1). However, his highest individual rating (8) was given for one of the trials of condition C3.

The final acoustic stimuli, among all trials, with the highest similarity rating selected by each subject were recorded (C2 for subjects S2 and S4, and C3 for subjects S1, S3, and S5), and are provided as audio files (Multimedia File S1, Multimedia File S2, Multimedia File S3, Multimedia File S4, Multimedia File S5 for subjects S1–S5, respectively), before the application of frequency-shaped amplification from the NAL-RP formula.

## Discussion

The three conditions chosen in this experiment aimed to investigate the perceptual characteristics of steady electric stimulation on electrode 22 of Cochlear® devices, by comparing such stimulation with a selected range of sound qualities. The first condition was intended to show whether the sound sensation was more similar to a white noise or a pure tone. The second condition was designed to explore the spectral shape of the sound in case of the selection, at the end of C1, of a sound that was not a pure tone. The third condition aimed to test a possible compression or expansion of the spacing between the frequency components of the acoustic signal. Because it has been suggested, from pitch-matching studies, that a reorganization of the auditory system may occur within the months following implantation [9], [10], the subjects of the current study were selected to have at least one year of CI use. It was supposed that the results obtained were representative of a stable sensation.

The experimental results from these 5 CI users suggest that the sound sensation produced by steady electrical stimulation on electrode 22 was not close to that of either a pure tone or a white noise. The data from condition C1 are consistent with the subjects generally perceiving the electrical stimulus as similar to a complex sound with an intermediate bandwidth (average Q factor of 20.2). This finding indicates that simulators using either noise-band carriers [2] or pure tones [6] may be inaccurate in representing the acoustic sensation corresponding to electric stimulation on a single, apical electrode. The data from C2, in which subjects adjusted the parameters of a harmonic complex tone, showed an average Q factor of 13.4. Figure S3B shows that subjects selected sounds with quite similar complexity. As mentioned above, each subject selected a similar center frequency for the bandpass filters in C1 and C2. This shows that, in Condition 2, subjects selected a signal with the maximum amplitude around the F0, and with a progressive decrease of the energy of the following components. In Condition 3, all average values of the inharmonicity exponent selected by the subjects were in the range of 1.1 to 1.7 (Figure S3C), corresponding to a moderate, progressive increase in the spacing between the frequency components of the acoustic signal.

In a previous study that estimated the pitch corresponding to steady stimulation on electrode 22 in a group of 14 bimodal users of Cochlear® devices, the average frequency of a pitch-matched pure tone was 483.6 Hz (range: 257.4–887.0 Hz) [10]. This may be compared to the subjects’ setting of the center frequency of the bandpass filter in condition C2 in the present study, as it may be assumed that this setting would dominate the pitch sensation. Note that the same bandpass filter setting was also applied in condition C3. The average center frequency was 523 Hz, which is similar to the average frequency of the pitch-matched tone reported previously. However, similarly to Green et al [24], a large inter- and intra-variability in pitch matching across subjects was observed, which seemed unrelated to the pure-tone thresholds of the non-implanted ear (Figure S1). In the cited study, the authors showed that subjects with similar audiograms displayed different degrees of frequency selectivity. Subjects able to produce consistent pitch matching were those who showed frequency selectivity extending beyond 500 Hz. In the present study, frequency selectivity may have varied across frequencies and among subjects, possibly affecting the pitch-matched frequencies. Additional potential effects on matching would have included the electrode position, which presumably would have differed between subjects as a consequence of differing surgical insertion depths.

The subjects’ ratings showed that condition C1 did not provide a satisfactory match. Conditions C2 and C3 were rated similarly on average. Nevertheless, subjects S2 and S4 rated higher C2 (harmonic complex sound), while subjects S3 and S5 gave a higher rate when an expansion was added across the frequency components. Clinically, subjects S2 and S4 were the two youngest and subject S2 had the best residual hearing.

Electrical stimulation via electrode 22 was chosen because its apical position was expected to correspond well to the cochlear region where these subjects had most residual hearing. However, all the subjects had impaired acoustic hearing in the ear used for the comparisons. Characteristics of an impaired cochlea, such as larger auditory-filter bandwidths and as a consequence lower frequency selectivity, as well as poorer sensitivity, may have resulted in these subjects’ perception of the acoustic signals [25]. The different rating of subject S2 may arise from a less impaired cochlea. The results obtained in the present study may differ from that expected with normal hearing. In particular, the progressive increase in the spacing between the frequency components may arise from the hearing impairments listed above.

In summary, the sound sensation created by stimulation on electrode 22 of CI recipients with abnormal residual hearing in the acoustically tested ear was most similar, out of the acoustic signals presented in these experiments, to that of a complex sound with a spectral envelope peak at approximately 523 Hz. For 3 subjects, the sound was more inharmonic with a progressive increase in the spacing between the frequency components. These results describe the characteristics of the sensation provided by a pulse train for subjects with contralateral residual hearing. However, generalization to other electrodes and places of stimulation is so far not possible. This study is a first step, and needs to be extended to other electrodes, to be studied over time, from the beginning of CI use, to evaluate the plasticity of the auditory pathways, and to be reproduced with implanted subjects presenting with single-sided deafness to evaluate the modifications induced by a hearing impaired cochlea on acoustic stimuli.

## Supporting Information

Figure S1 **Hearing threshold levels for the non-implanted ear in each subject.**
(TIF)

Figure S2 **Sketch of the experimental set-up** **(A) and of** **the 3 experimental conditions (B).** Each subject was asked to compare an electric stimulus with an adjustable acoustic stimulus using a graphical interface (A, left of diagram), as described in the text. The right part of the diagram (B) shows the parameters that were adjusted by the subjects, and the corresponding spectra of the acoustic signals.
(TIF)

Figure S3 **Mean results for each of the subjects (left panels) and representative spectra for the corresponding sounds (right panels) in each of the 3 conditions (panels A-C).** The horizontal and vertical cross-hairs represent the standard deviations of responses provided by each subject. The sizes of the circles represent each subject’s mean similarity rating; i.e., how similar the acoustic sound was to the electric sensation, larger circles indicating closer similarity.
(TIF)

Table S1 Relevant characteristics of the subjects and their CI systems.
(DOC)

Multimedia File S1 **Final acoustic signal with the highest similarity rating from C3 for subject S1.**
(WAV)

Multimedia File S2 **Final acoustic signal with the highest similarity rating from C2 for subject S2.**
(WAV)

Multimedia File S3 **Final acoustic signal with the highest similarity rating from C3 for subject S3.**
(WAV)

Multimedia File S4 **Final acoustic signal with the highest similarity rating from C2 for subject S4.**
(WAV)

Multimedia File S5 **Final acoustic signal with the highest similarity rating from C3 for subject S5.**
(WAV)
