# Supplementary figures and images for: The Sound Sensation of Apical Electric Stimulation in Cochlear Implant Recipients with Contralateral Residual Hearing
Source: PLoS One. 2012 Jun 19;7(6):e38687. doi: 10.1371/journal.pone.0038687 (PMC3378545; doi:10.1371/journal.pone.0038687)

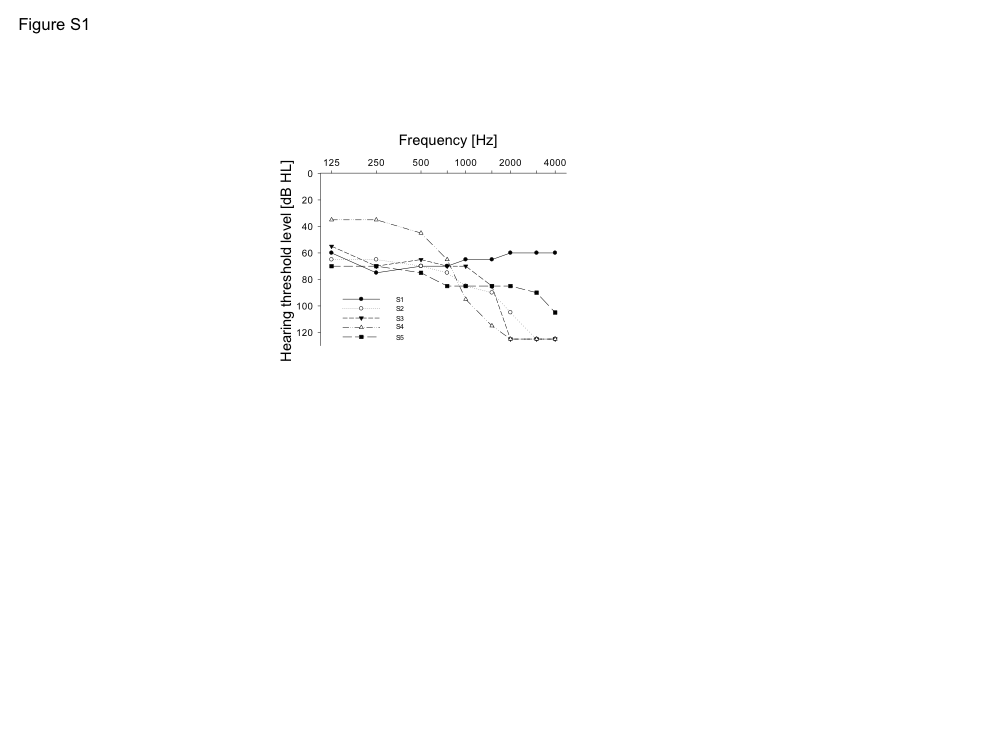

Supplement: Figure S1 — Hearing threshold levels for the non-implanted ear in each subject. (TIF) [file pone.0038687.s001.tif]

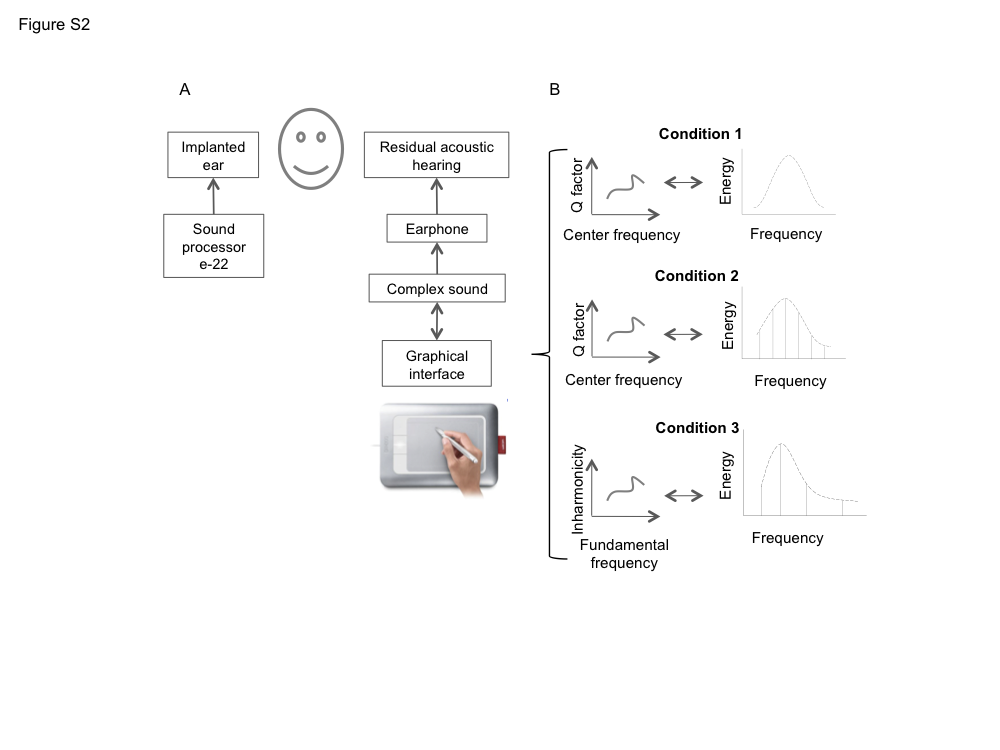

Supplement: Figure S2 — Sketch of the experimental set-up (A) and of the 3 experimental conditions (B). Each subject was asked to compare an electric stimulus with an adjustable acoustic stimulus using a graphical interface (A, left of diagram), as described in the text. The right part of the diagram (B) shows the parameters that were adjusted by the subjects, and the corresponding spectra of the acoustic signals. (TIF) [file pone.0038687.s002.tif]

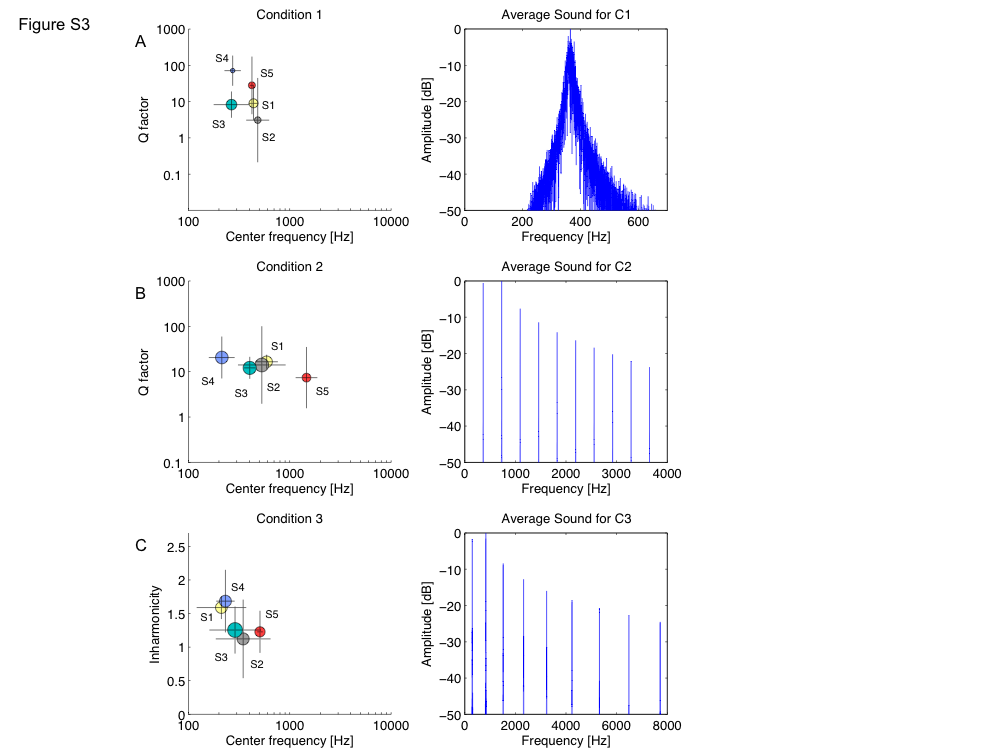

Supplement: Figure S3 — Mean results for each of the subjects (left panels) and representative spectra for the corresponding sounds (right panels) in each of the 3 conditions (panels A-C). The horizontal and vertical cross-hairs represent the standard deviations of responses provided by each subject. The sizes of the circles represent each subject’s mean similarity rating; i.e., how similar the acoustic sound was to the electric sensation, larger circles indicating closer similarity. (TIF) [file pone.0038687.s003.tif]
