# Supplementary material for: The Sound Sensation of Apical Electric Stimulation in Cochlear Implant Recipients with Contralateral Residual Hearing
Source: PLoS One. 2012 Jun 19;7(6):e38687. doi: 10.1371/journal.pone.0038687 (PMC3378545; doi:10.1371/journal.pone.0038687)
Supplement: Table S1 — Relevant characteristics of the subjects and their CI systems. (DOC) [file pone.0038687.s004.doc]

| **Subject** | **Sex** | **Age (y)** | **Etiology** | **CI processor** | **Rate (Hz)** | **CI use (y)** | **CNC word recognition (% correct)** |
| --- | --- | --- | --- | --- | --- | --- | --- |
| S1 | F | 78 | Unknown | Freedom | 900 | 1.5 | 61 |
| S2 | F | 47 | Viral | Freedom | 900 | 2 | 50 |
| S3 | M | 68 | Acoustic trauma | Esprit3G | 700 | 6 | 74 |
| S4 | F | 60 | Unknown | Freedom | 900 | 1.9 | 76 |
| S5 | M | 62 | Unknown | CP810 | 900 | 1.1 | 55 |
